# A simultaneous multiple angle-wavelength dispersive X-ray reflectometer using a bent-twisted polychromator crystal

**DOI:** 10.1107/S0909049512043415

**Published:** 2012-11-15

**Authors:** Tadashi Matsushita, Etsuo Arakawa, Wolfgang Voegeli, Yohko F. Yano

**Affiliations:** a Photon Factory, Institute of Materials Structure Science, KEK, Tsukuba, Ibaraki, Japan; b Department of Physics, Tokyo Gakugei University, Koganei, Tokyo, Japan; c Department of Physics, Kinki University, Higashiosaka, Osaka, Japan

**Keywords:** X-ray reflectivity curve, time-resolved measurement, bent-twisted polychromator crystal, simultaneous measurement

## Abstract

An X-ray reflectometer has been developed, which can simultaneously measure the whole specular X-ray reflectivity curve with no need for rotation of the sample, detector or monochromator crystal during the measurement. A bent-twisted crystal polychromator is used to realise a convergent X-ray beam which has continuously varying energy *E* (wavelength λ) and glancing angle α to the sample surface as a function of horizontal direction. This convergent beam is reflected in the vertical direction by the sample placed horizontally at the focus and then diverges horizontally and vertically. The normalized intensity distribution of the reflected beam measured downstream of the specimen with a two-dimensional pixel array detector (PILATUS 100K) represents the reflectivity curve. Specular X-ray reflectivity curves were measured from a commercially available silicon (100) wafer, a thin gold film coated on a silicon single-crystal substrate and the surface of liquid ethylene glycol with data collection times of 0.01 to 1000 s using synchrotron radiation from a bending-magnet source of a 6.5 GeV electron storage ring. A typical value of the simultaneously covered range of the momentum transfer was 0.01–0.45 Å^−1^ for the silicon wafer sample. The potential of this reflectometer for time-resolved X-ray studies of irreversible structural changes is discussed.

## Abstract 2

Using a convergent X-ray beam having continuously varying energy and glancing angle as a function of direction, the whole profile of a specular X-ray reflectivity curve is measured with no need for any mechanical motion during the measurement.

## 1. Introduction

Specular X-ray reflectometry is an established structural probe for characterizing the surfaces and interfaces of materials (Daillant & Gibaud, 1999 ▶). By analyzing X-ray reflectivity curves, it is possible to determine the thickness, electron density distribution along the surface normal, and surface and interfacial roughness of thin films and multilayers. A popular method of measuring X-ray reflectivity curves is the angle-dispersive method using collimated and monochromatic X-rays, where the reflected intensity measurement is repeated many times by successively changing the glancing angle of X-rays to the sample surface. Another method is the energy-dispersive method, where a white X-ray beam is used in combination with an energy-dispersive solid state detector with a fixed glancing angle of X-rays. The data collection time for the angle-dispersive method is typically minutes to tens of minutes even using intense synchrotron radiation and that for the energy-dispersive method is seconds to minutes (Bhattacharya *et al.*, 2003 ▶). Until recently, most time-resolved X-ray reflectivity measurements have been limited to time scales of minutes to hours (Richter *et al.*, 1998 ▶; Rossi Abertini *et al.*, 2003 ▶; Generosi *et al.*, 2005 ▶; Paci *et al.*, 2005 ▶, 2006 ▶; Gonzalez-Silveria *et al.*, 2007 ▶; Yano *et al.*, 2009 ▶). Millisecond time-resolution X-ray reflectometers were proposed (White *et al.*, 1999 ▶; Garrett *et al.*, 2001 ▶), in which white X-rays reflected by the sample are simultaneously wavelength-dispersed by single or multiple crystal analyzer(s). An exceptionally high time resolution of 100 ps was achieved recently by Nüske *et al.* (2011 ▶) in a pump–probe mode with the angle-dispersive method. However, in this pump–probe mode the structural changes of the sample must be repeated many thousands of times at every glancing angle position of the sample, thus this method is not applicable to samples in which structural changes are irreversible or not easily repeatable. For studying the structural kinematics or dynamics of such samples, it would be better to be able to simultaneously measure the whole profile of the X-ray reflectivity curve and to successively measure the time-dependent changes after applying a stimulus to the specimen.

In previous studies (Matsushita *et al.*, 2008 ▶, 2009 ▶, 2010 ▶) we reported a method of simultaneously measuring specular X-ray reflectivity curves in milliseconds to seconds with no need for angle scanning of the sample, detector or polychromator crystal during measurement. In this method the reflectivity curve is measured with a position-sensitive detector as a function of X-ray energy using a convergent X-ray beam which has a one-to-one correspondence between direction and energy. Although the data collection time was short, the practically measured range of the perpendicular momentum transfer was not wide enough because the X-ray energy range was limited. Moreover, the minimum detectable reflectivity was also limited to approximately 10^−6^ because of the read-out noise of the X-ray CCD detector.

In this study we report improvements of the method to widen the simultaneously covered range of the perpendicular momentum transfer, *Q*, and to lower the detectable minimum reflectivity. For widening the *Q*-range, we used a convergent X-ray beam for which both the energy *E* and the glancing angle α to the sample surface change continuously as a function of direction. We kept the geometry such that the sample surface was stationary in the horizontal plane in order to be able to study liquid surfaces. For lowering the detectable minimum reflectivity, we used a photon-counting pixel array detector with no read-out noise and a very sharp point-spread function.

## 2. Principle of the method

### 2.1. X-ray optics and simultaneously covered range of momentum transfer

A specular X-ray reflectivity curve is a plot of the X-ray reflectivity as a function of the perpendicular momentum transfer *Q* which is defined by
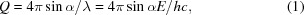
where α is the glancing angle of X-rays, λ is the wavelength, *E* is the energy, *h* is the Planck constant and *c* is the speed of light.

**Figure 1:**
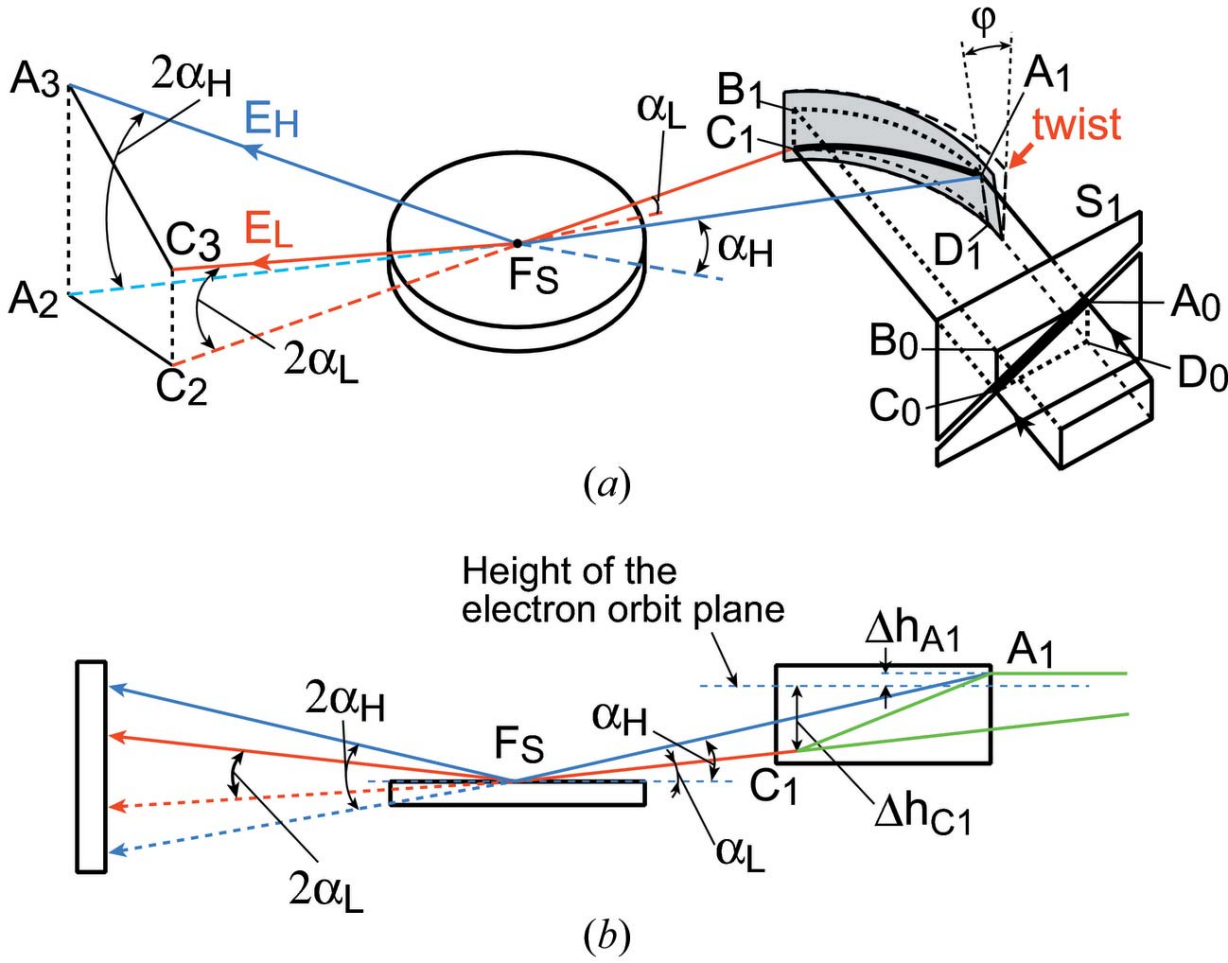
Bird’s eye (*a*) and side (*b*) views of the geometry of the simultaneous multiple angle-wavelength dispersive X-ray reflectometer. The crystal is bent horizontally to realise horizontal focusing and then twisted in such a way that the surface normal along the straight line *A*_1_ *D*_1_ is inclined downward by an angle ϕ, while that along *B*_1_ *C*_1_ stays horizontal in the original direction. α_H_ is the glancing angle of the ray along the path *A*_1_ *F*_S_ to the sample surface at *F*_S_, and α_L_ is that for the ray along the path *C*_1_ *F*_S_. The X-ray beam is specularly reflected by a sample placed horizontally at *F*_S_, then horizontally diverges directed toward points on the line *A*_3_ *C*_3_ on the detector surface.

The geometry of the present method is schematically shown in Fig. 1 ▶. The main component of the X-ray optics is the curved crystal polychromator in the reflection geometry. The diffracting plane of the polychromator crystal is assumed to be parallel to the surface. A synchrotron white X-ray beam from a bending-magnet source is incident on the polychromator crystal. The horizontal and vertical beamline slits are kept wide open to guide an X-ray beam having a rectangular cross section *A*_0_ *B*_0_ *C*_0_ *D*_0_ into the experimental hutch. An inclined slit is placed upstream of the polychromator crystal so that a beam through one end of the slit aperture hits the upper-right corner of the polychromator crystal at *A*_1_, which is slightly (1–3 mm) above the electron orbit plane of the storage ring by an amount Δ*h*_*A*1_, and a beam through the other end of the slit aperture hits the lower-left corner of the crystal at *C*_1_, which is below the electron orbit plane by an amount Δ*h*_*C*1_ (10–15 mm).

**Figure 2:**
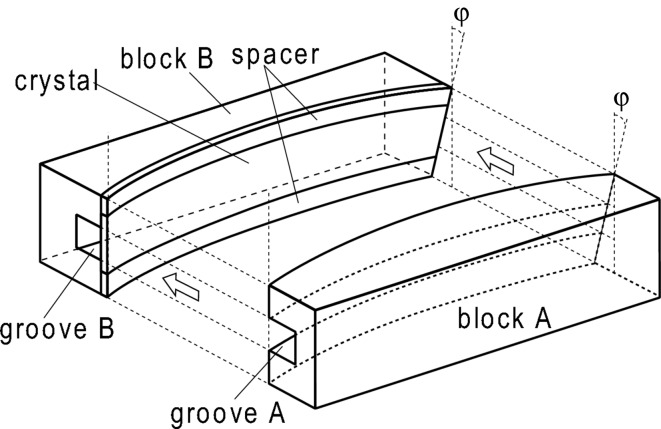
A bender used to create the bent-twisted crystal polychromator. The crystal was bent and twisted by sandwiching it between two water-cooled bronze blocks which have preconfigured convex and concave surfaces.

If the crystal is ellipsoidally bent such that the X-ray source *S* and the focal point *F*_S_ are located at its two foci, all the X-ray beams reflected by the crystal are directed toward the focus *F*_S_. However, we do not use a crystal with an ellipsoidal shape because it is difficult to bend the crystal to an ellipsoidal shape. Instead, we used a bent and twisted crystal. We first calculated the surface normal directions along the curve *A*_1_ *C*_1_ of an ellipsoid. Next, we considered a surface that consists of the tangents to a generating line of the ellipsoid at each point of the curve *A*_1_ *C*_1_. The resulting surface is a train of almost vertical straight lines with gradually changing direction that touch the ellipsoid on the curve *A*_1_ *C*_1_. We then prepared concave and convex bronze blocks which mimic such a surface. The thin crystal is sandwiched between these convex and concave shaped surfaces, as is shown in Fig. 2 ▶. An X-ray beam having infinitesimally small vertical width incident exactly on the curve *A*_1_ *C*_1_ is focused at *F*_S_. If the vertical width of the inclined slit *S*_1_ upstream of the polychromator crystal is finite, but small enough, X-ray beams reflected at points near the curve *A*_1_ *C*_1_ are horizontally focused and vertically condensed to a tiny spot at *F*_S_. The direction of the surface normal at any point along the straight line *A*_1_ *D*_1_ is inclined downward by an angle ϕ, while that at any point along the vertical line *B*_1_ *C*_1_ stays horizontal. The beam diffracted at *C*_1_ keeps the original vertical deflection angle and is directed toward *F*_S_. The vertical deflection angle from the horizontal plane of the beam along the line *C*_1_ *F*_S_ is the same as that of the beam along *C*_0_ *C*_1_ and is given by
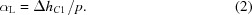
Here, *p* is the distance from the polychromator crystal to the X-ray source. The beam diffracted at *A*_1_ is deflected downward by an angle δ_H_ defined by
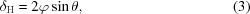
where θ is the Bragg angle at *A*_1_. The angle between the line *A*_1_ *F*_S_ and the horizontal plane is given by
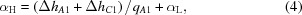
where *q*_*A*1_ is the distance from the focus *F*_S_ to point *A*_1_. By setting the twist angle ϕ of the crystal to realise the relation δ_H_ = α_H_, the beam reflected at *A*_1_ is directed toward *F*_S_. At the same time the beam diffracted at an arbitrary point *P* (not shown in Fig. 1 ▶) along *A*_1_ *C*_1_ is also directed toward *F*_S_. The beam represented by the line *A*_0_ *C*_0_ is horizontally focused and vertically condensed at *F*_S_ after being reflected by the crystal.

The sample is placed almost horizontally at *F*_S_. The X-ray beam is specularly reflected in the vertical direction. The glancing angle α of each ray of the convergent X-ray beam continuously varies between α_L_ and α_H_ depending on its horizontal path direction. Downstream of the focus, the X-ray beam diverges horizontally and directs towards points on the line *A*_3_ *C*_3_ on the detector surface. At the same time, the energy *E* (wavelength λ) of the X-ray beam reflected at *P* also continuously varies between *E*_H_ and *E*_L_ along the line *A*_1_ *C*_1_, where *E*_H_ and *E*_L_ are the energies of the X-ray beams monochromated at *A*_1_ and *C*_1_, respectively.

The perpendicular momentum transfer for the ray from *P* to *F*_S_ is given by equation (1), resulting in a continuous change of the perpendicular momentum transfer along *A*_3_ *C*_3_. α and *E* can be experimentally determined as will be described in §3.1. When the sample is removed, the X-ray beam hits points on the line *A*_2_ *C*_2_ on the detector surface. By normalizing the reflected intensity distribution *I* measured along the line *A*_3_ *C*_3_ by the intensity distribution *I*_0_ along the line *A*_2_ *C*_2_, the X-ray reflectivity curve profile is obtained without any mechanical movement of the sample, detector or polychromator crystal.

### 2.2. Resolution

The momentum transfer resolution of the system at the X-ray energy *E* is dependent on the energy spread δ*E* and the vertical angular spread δα of the X-ray beam detected by each pixel element of the detector. The spread in the momentum transfer caused by the energy spread of the X-ray beam is given by the relation δ*Q*_*E*_/*Q* = δ*E*/*E*. The latter is simply given by δ*Q*/*Q* = δαcotα, where α is the angle between the convergent X-ray beam and the surface of the sample.

The energy spread can be characterized into the four contributions δ*E*_1_, δ*E*_2_, δ*E*_3_ and δ*E*_4_, which come from (1) the horizontal size of the source, (2) the size of a detector pixel, (3) the thickness of the polychromator crystal and (4) the intrinsic angular width of diffraction, respectively. The contribution from the thickness of the crystal comes from the fact that the X-ray beam penetrates into the crystal and is diffracted within the crystal. These four factors can roughly be estimated by simple ray-tracing calculations. When we assume a 0.3 mm-thick Si (111) crystal in the reflection geometry, *p* = 20.5 m, crystal-to-focus distance = 0.43 m, a detector pixel size of 0.172 mm, specimen-to-detector distance = 580 mm and horizontal size of the source = 3.0 mm, it is roughly estimated that δ*E*_1_ ≃ 13 eV, δ*E*_2_ ≃ 15 eV, δ*E*_3_ ≃ 5 eV and δ*E*_4_ ≃ 3 eV at ∼19 keV. The total energy spread δ*E* = (δ*E*_1_^2^ + δ*E*_2_^2^ + δ*E*_3_^2^ + δ*E*_4_^2^)^1/2^ can be estimated to be approximately 21 eV, resulting in δ*Q*_*E*_/*Q* = δ*E*/*E* = 1.1 × 10^−3^.

Two factors should be considered regarding the angular resolution. One is the angular divergence δα_1_ of the X-ray beam of an infinitely small horizontal width. Since the surface normal does not change along a vertical line of the polychromator crystal, the angular divergence of this X-ray beam is determined by the vertical width of the slit δ_w_, the vertical size δ_sv_ of the source and the source-to-slit distance *p*_S–S_. If δ_w_ = 0.1 mm, δ_sv_ = 0.2 mm and *p*_S–S_ = 20.5 m, δα_1_ is estimated to be 1.1 × 10^−3^ degrees (2 × 10^−5^ radians), which is sufficiently small. Another factor is the scattering angle covered by a single pixel element of the detector, which is given by
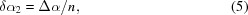
where Δα = α_H_ − α_L_, and *n* is the number of pixel elements which are required to cover this angle range. If Δα is 1.5° and *n* is 150, the angle resolution is 0.01° (1.75 × 10^−4^ rad). This gives a resolution δ*Q*_α_/*Q* = δα_2_cotα. For α = 1.0°, this value is 0.01.

With such estimations it is understood that the resolution of the momentum transfer is mainly governed by the angular resolution of the system in the present geometry.

### 2.3. Intensity of the reflected X-ray beam

**Figure 3:**
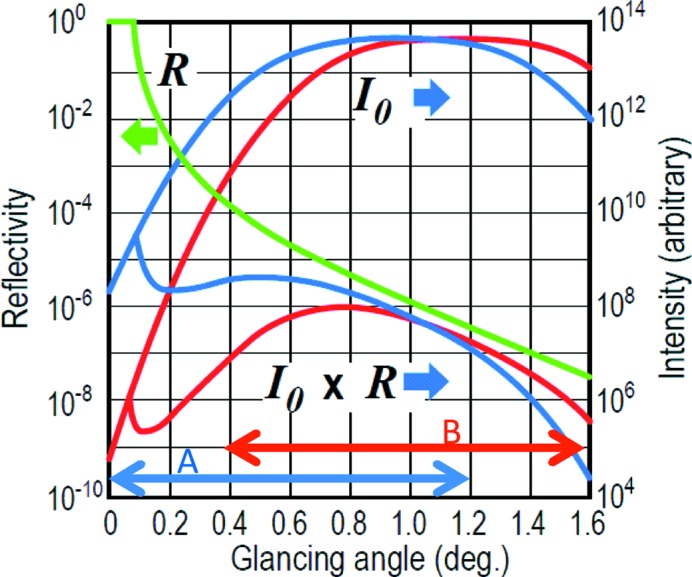
A semi-quantitative representation of the distribution of the specularly reflected beam intensity as a function of the glancing angle of the X-ray beam to the sample surface. *I*_0_: intensity distribution of the convergent X-ray beam incident onto the sample. Blue and red lines are for cases α_0_ = 0.96° and α_0_ = 1.20°, where α_0_ is the glancing angle which the horizontal X-ray beam from the source to the polychromator crystal makes with the sample surface after being reflected downward by the polychromator crystal. *R*: the specular reflectivity of a sample (silicon single crystal). *I*_0_ × *R*: the product of *I*_0_ and *R* which approximately represents the intensity distribution *I* of the X-ray beam specularly reflected by the sample.

The intensity of the synchrotron X-ray beam diffracted at *C*_1_ is several orders of magnitude weaker than that at *A*_1_ because the height at *A*_1_ is much closer to that of the electron orbit plane than the height at *C*_1_ as is shown in Fig. 1(*b*) ▶. The intensity of the diffracted X-ray beam along the line *A*_1_ *C*_1_ is given in Fig. 3 ▶, where the abscissa indicates the angle of the ray from a point on the line *A*_1_ *C*_1_ to *F*_S_. Since the energy (*E*_H_) of the beam diffracted at *A*_1_ is only 15–20% higher than that (*E*_L_) of the beam diffracted at *C*_1_ in the present geometry, as will be described in §3.1, we neglect the energy dependences of the bending-magnet radiation intensity, the Bragg reflectivity and the sensitivity of the detector in the semi-quantitative discussion of the observed reflected intensity. *I*_0_ is the intensity distribution of the X-ray beam after reflection by the polychromator crystal for two cases, α_0_ = 0.96° or 1.20°. Here, α_0_ is the glancing angle that the horizontal X-ray beam from the source to the polychromator crystal makes with the sample surface after being reflected downward by the polychromator crystal. *I*_0_ is proportional to the vertical intensity distribution along the line connecting *A*_1_ to *D*_1_ within the approximation described above. *I*_0_ is calculated using the calculation code *SPECTRA* (Tanaka & Kitamura, 2001 ▶) for the case of the bending-magnet radiation of the 6.5 GeV ring (horizontal emittance = 294 nmrad) of the Photon Factory. The value of α_0_ can be controlled by adjusting the vertical inclination of the polychromator crystal. The covered glancing angle range for the beams from *C*_1_ to *A*_1_ is shown by the two-way arrows *A* and *B* for these two cases. The positions shown by the left ends of the two-way arrows *A* and *B* correspond to the glancing angles of the X-ray beams through paths *C*_1_ *F*_S_ and the right ends to those of the X-ray beams through the path *A*_1_ *F*_S_. The curve *R* is the specular reflectivity of the sample. The specular reflectivity of the sample for the beam along *A*_1_ *F*_S_ is several orders of magnitude lower than that for the beam along *C*_1_ *F*_S_. The reflected beam intensity distribution along *A*_3_ *C*_3_ on the detector surface is proportional to the product *I*_0_ × *R*. Note that the difference between maximum and minimum intensities of the curve *I*_0_ × *R* is within only three or four orders of magnitude in spite of the difference of seven to eight orders of magnitude in *R*. This makes time-resolved measurements much easier.

## 3. Experiments

### 3.1. X-ray optics

As a polychromator crystal, a silicon (111) wafer of size 200 mm × 20 mm × 0.3 mm was used in the reflection geometry. To bend and twist the crystal, we prepared two directly water-cooled bronze metal blocks having convex and concave surfaces as discussed in §2.1 and shown in Fig. 2 ▶. Moreover, we made grooves in the middle parts of these bronzes: groove *A* was made to secure incident and reflected X-ray beam paths and groove *B* was to remove any scattering materials right behind the crystal and to lower the background intensity. The crystal was sandwiched between these convex and concave surfaces with spacers to reduce clamping forces acting on the crystal as shown in Fig. 2 ▶. The thermal contact between the bronze blocks and the crystal was kept by using liquid gallium–indium alloy. Without the water cooling or in the case where the water cooling was insufficient, the shape of the diffracted X-rays was deformed suggesting thermal deformation of the crystal. The shapes of these concave and convex surfaces were designed and configured so as to cause horizontal focusing and vertical condensing of reflected X-rays at 430 mm from the center of the crystal when the source-to-crystal distance is 20.5 m and the Bragg angle for the central beam is 6.28°. The slit upstream of the polychromator crystal was 1.0 mm wide and was inclined 36.4° from the horizontal line, so the shape of the X-ray beam incident on the polychromator crystal is the diagonal line of a rectangle [16.6 mm (H) × 12.2 mm (V)]. Downstream of the polychromator, the shape of the X-ray beam was a slightly curved and inclined line with a width of 1 mm. An inclined straight-line slit with a width of 0.1 mm was placed downstream of the polychromator crystal so that the intensity distribution of the X-ray beam incident on the sample is along a linear line downstream of this slit. A slit and a helium beam path covered with lead sheets were also placed downstream of the sample to reduce background intensities.

The beam size at the focus position was observed by a two-dimensional pixel array detector [PILATUS 100K (Kraft *et al.*, 2009 ▶)] with appropriate beam absorbers which made the intensity distribution along the inclined line relatively flat in order to bring the intensities of any parts of the X-ray beam within the dynamic range of the detector and to image the focus with relatively equal contributions from different energy components. By such an observation, the horizontal focus size was measured to be 0.8 mm at a distance of 430 mm from the center of the polychromator crystal. The vertical X-ray beam spot size was measured to be 0.2 mm also at a distance of 430 mm by vertically scanning a knife edge with the PILATUS detector located 580 mm downstream of the focus and differentiating the obtained intensity distribution.

To measure the intensity distribution of the specularly reflected beam, the PILATUS 100K detector was placed 580 mm downstream of the specimen. The sensitive area of the detector is 83.8 mm × 33.5 mm and the size of a pixel element is 172 µm × 172 µm. The energy of the X-ray beam was calibrated by observing the positions of absorption edges at the detector surface when several kinds of metal foils were placed at the focus position in a similar manner as reported earlier (Matsushita *et al.*, 2007 ▶). The energy range was from around 16 keV to 21 keV along the line *A*_1_ *C*_1_ of the polychromator crystal shown in Fig. 1 ▶. For example, in the case of the silicon single-crystal sample described in §3.2 and §4.1 the X-ray energy varied from 16.9 keV to 20.3 keV. The energy spread covered by one pixel was estimated to be 24.1 eV by dividing the whole covered energy range (16.9 keV to 20.3 keV) by the number (141) of pixels to cover this energy range.

The position of the reflected beam was easily recorded by the detector without using any absorber, while the position of the direct beam without the specimen was recorded with several absorbers in order to avoid saturation of pixel elements of the detector. With such measurements, the vertical separation between the reflected and direct beams was measured as a function of the horizontal position on the detector surface. By dividing halves of these vertical separations with the distance between the X-ray beam focus and the detector, glancing angles of the X-ray beam were determined as a function of the horizontal position on the detector surface. In the case of silicon crystal samples (§3.2), the glancing angle varied from 0.033° to 1.24° within the convergence of the X-ray beam.

### 3.2. Samples

Samples were placed almost horizontally at the focus position. The glancing angle of the X-ray beam could be controlled by rotating the sample around the horizontal rotation axis except in the case of liquid samples. As samples, we used a commercially available mechano-chemically polished (100) silicon wafer, a 15.4 nm-thick gold film coated on a silicon single-crystal substrate and liquid ethylene glycol. The mechano-chemically polished silicon sample was studied to check whether a wide *Q*-range could be covered simultaneously and to determine the detectable minimum reflectivity. The gold film sample was used in order to check whether Kiessig fringes (Kiessig, 1931 ▶) could be observed. The size of the substrate was 15 mm × 10 mm × 0.5 mm. The ethylene glycol sample filled in a shallow 100 mm-diameter circular trough was used to demonstrate that a liquid surface can be studied with the present reflectometer. No anti-vibration devices were installed when the sample was measured.

### 3.3. Measurements of intensity and position of specularly reflected X-ray beam

**Figure 4:**
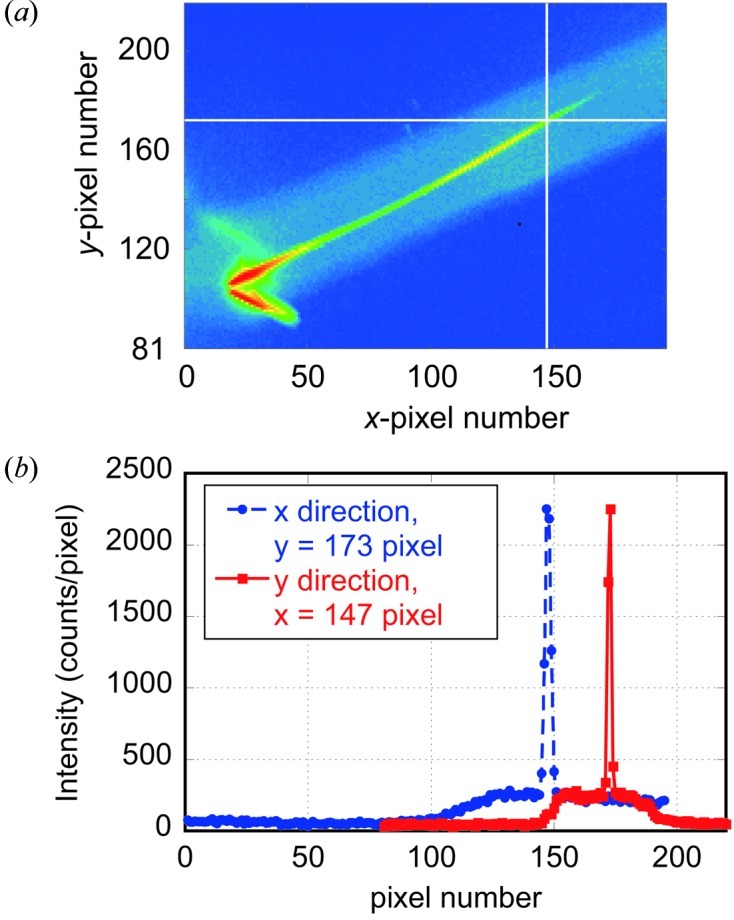
(*a*) Part of the detector image of the X-ray beam reflected from the silicon single-crystal sample. The *x*-axis is parallel to the horizontal direction across the X-ray beam direction, while the *y*-axis is parallel to the vertical direction. Units on the *x*- and *y*-axes are pixel numbers of the detector and the size of a pixel is 172 µm × 172 µm. The dark parts are shadows of a slit downstream of the sample. The inclined broad and slightly bright band represents X-ray intensity coming through the slit downstream of the sample and this consists of coplanar and non-coplanar diffuse scattering. A much brighter inclined line in the middle of the broad band is the specularly reflected beam. (*b*) Intensity profiles along white lines parallel to *x* (blue) and *y* (red) axes in (*a*) including a specular reflection at *Q* = 0.4 Å^−1^.

The intensity distribution *I* of the reflected beam from the sample was measured by the PILATUS detector placed 580 mm downstream of the focus. Fig. 4 ▶ shows an example of a part of the recorded detector output image of the specularly reflected beam from the silicon wafer sample. The *x*- and *y*-axes are parallel to the horizontal and vertical directions, respectively. From the *y*-coordinate of the specularly reflected beam, the scattering angle at a particular horizontal position *x* can be determined. The *x*-coordinate can be converted to X-ray energy from the calibrated curve. From this X-ray energy and the scattering angle, the perpendicular momentum transfer *Q* was determined following equation (1). The intensity of the specularly reflected beam at each *Q* was obtained by plotting the intensity profile along the *y*-axis as is shown in Fig. 4(*b*) ▶ and then separating the specular reflection intensity from the background intensity. This problem of background subtraction from experimental data will further be discussed in §5.3.

### 3.4. Determination of the X-ray beam intensity distribution incident to the sample

To experimentally determine the reflectivity, it is also necessary to measure the intensity distribution *I*_0_ at the detector when the sample is removed from the X-ray path. The ratio between *I* and *I*_0_ gives the reflectivity *R*.

The difference between maximum and minimum intensities in *I*_0_ is six to seven orders of magnitude. Some pixel elements receiving X-ray beams in the direction near the electron orbit plane are easily saturated or the dead time of the detector becomes serious (Trueb *et al.*, 2012 ▶) even when using absorbers to attenuate the beam intensity. Moreover, the contribution from higher harmonics was enhanced when using absorbers, leading to erroneous estimation of *I*_0_. The following method was used to avoid these problems. In the region where *Q*(α) is small, the incident beam intensity *I*_0_ is low but the reflectivity is relatively high as is shown in Fig. 3 ▶. Both the reflected and direct beam intensities, *I* and *I*_0_, were measured without saturation of the detector even with no absorbers. The reflectivity *R* was determined from measured *I* and *I*_0_ in the corresponding horizontal range Δ*x*_1_ of the detector. Next, the sample was rotated slightly, with the result that the same *Q*-range was covered on the detector in its horizontal position range Δ*x*_2_ adjacent to Δ*x*_1_. Since *R* in this *Q*-range was already determined and the reflected beam intensity *I* in this range was measured, the incident beam intensity could be determined from the relation *I*_0_ = *I*/*R*. A typical count rate of *I* per pixel element was ten to several thousand per second which was sufficiently low and almost free from saturation of the detector. By repeating this procedure we determined the direct beam intensity distribution *I*_0_ across the surface of the detector.

## 4. Results

### 4.1. Reflectivity curves from a silicon (100) single-crystal wafer

**Figure 5:**
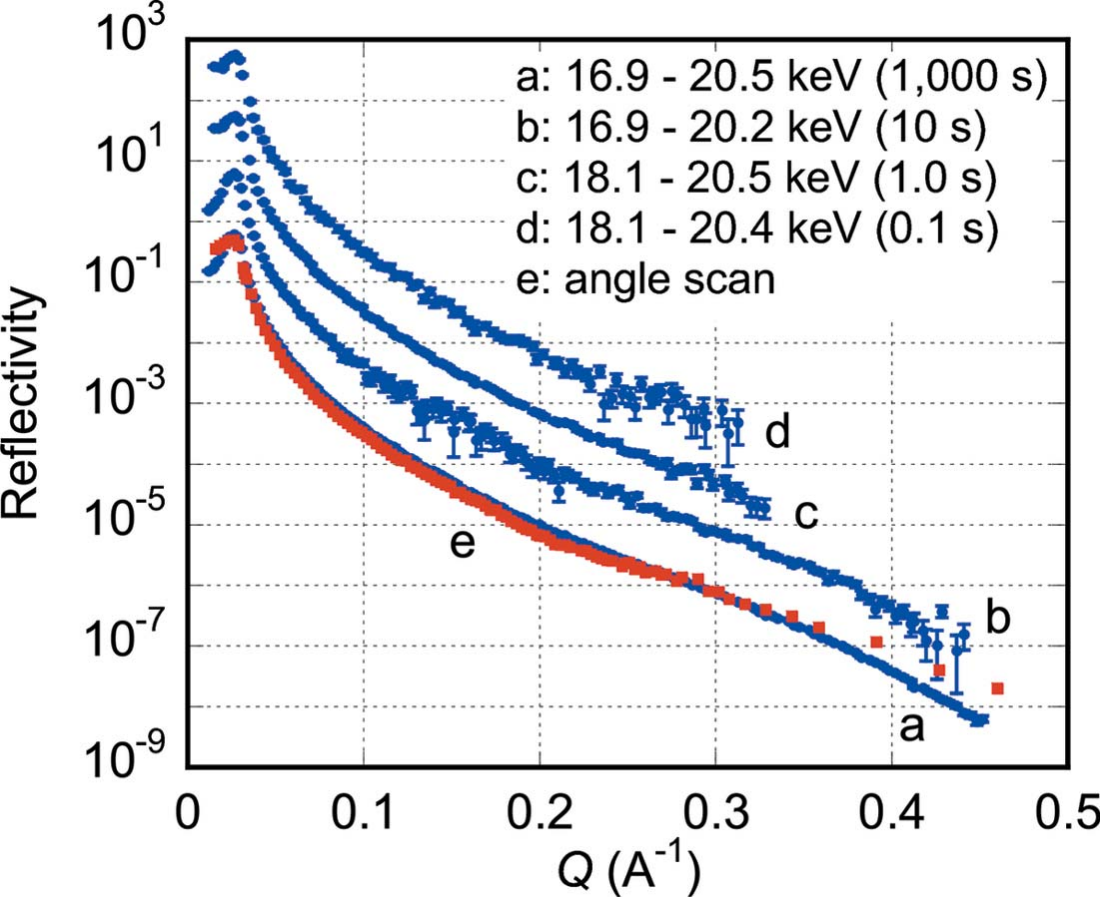
X-ray reflectivity curves from a mechano-chemically polished silicon (100) wafer. Curves *a* and *b* were obtained with data collection times of 1000 and 10 s, respectively. Curves *c* and *d* were obtained with data collection times of 1.0 and 0.1 s, respectively, after rotating the sample to lower the glancing angle in order to enhance the incident beam intensity *I*_0_ in the covered *Q*-range to compensate for the short exposure time. Each curve is shifted vertically for clarity. The used X-ray energy ranges are given in the figure. Curve *e* was obtained by the angle-scan mode using the present reflectometer setting with a horizontal slit of 0.1 mm width downstream of the polychromator. The angle-scan curve *e* was obtained by connecting seven partial reflectivity curves measured using seven different X-ray energies (17.7, 18.2, 18.6, 19.1, 19.5, 19.9 and 20.3 keV). Error bars are shown for all data points of all the curves.

Fig. 5 ▶ shows specular X-ray reflectivity curves from the silicon (100) wafer. Curve *a* was obtained with a data collection time of 1000 s. The reflectivity curve profile is recorded in the momentum transfer range from 0.01 to 0.45 Å^−1^. Typical signal count rates were 4300, 20 and 2.0 counts s^−1^ at *Q* = 0.02, 0.30 and 0.45 Å^−1^, respectively. The minimum reflectivity in curve *a* was 6.3 × 10^−9^ at *Q* = 0.45 Å^−1^. Curves *b*–*d* were obtained with data collection times of 10, 1.0 and 0.1 s, respectively.

Curve *e* was obtained by the angle-scan mode using the present reflectometer setting; the horizontal width of the X-ray beam was narrowed to 0.1 mm with an extra slit downstream of the polychromator and the glancing angle of the X-ray beam was scanned by rotating the sample around a horizontal axis. The vertical scale was taken to be the same as that for the curve *a*. In this measurement the measured *Q*-range was divided into seven regions. We placed a 0.1 mm-wide vertical slit at seven corresponding different horizontal positions to be able to measure the reflected intensity at a reasonable count rate. For example, at low *Q*-range, we placed the slit to select a low-intensity part of the convergent X-ray beam because the reflectivity from the sample is high, while at high *Q*-value we placed the slit to select a high-intensity part because the reflectivity from the sample is low. In this way we collected seven partial profiles of the reflectivity curve with seven slightly different X-ray energies (17.7, 18.2, 18.6, 19.1, 19.5, 19.9 and 20.3 keV) and connected these seven partial reflectivity profiles to make one profile, which is the curve *e*. In other words, the lower *Q*-part of curve *e* was measured with lower X-ray energies and the higher *Q*-part was measured with higher X-ray energy.

### 4.2. Reflectivity curves from a thin gold layer on a silicon single-crystal substrate

**Figure 6:**
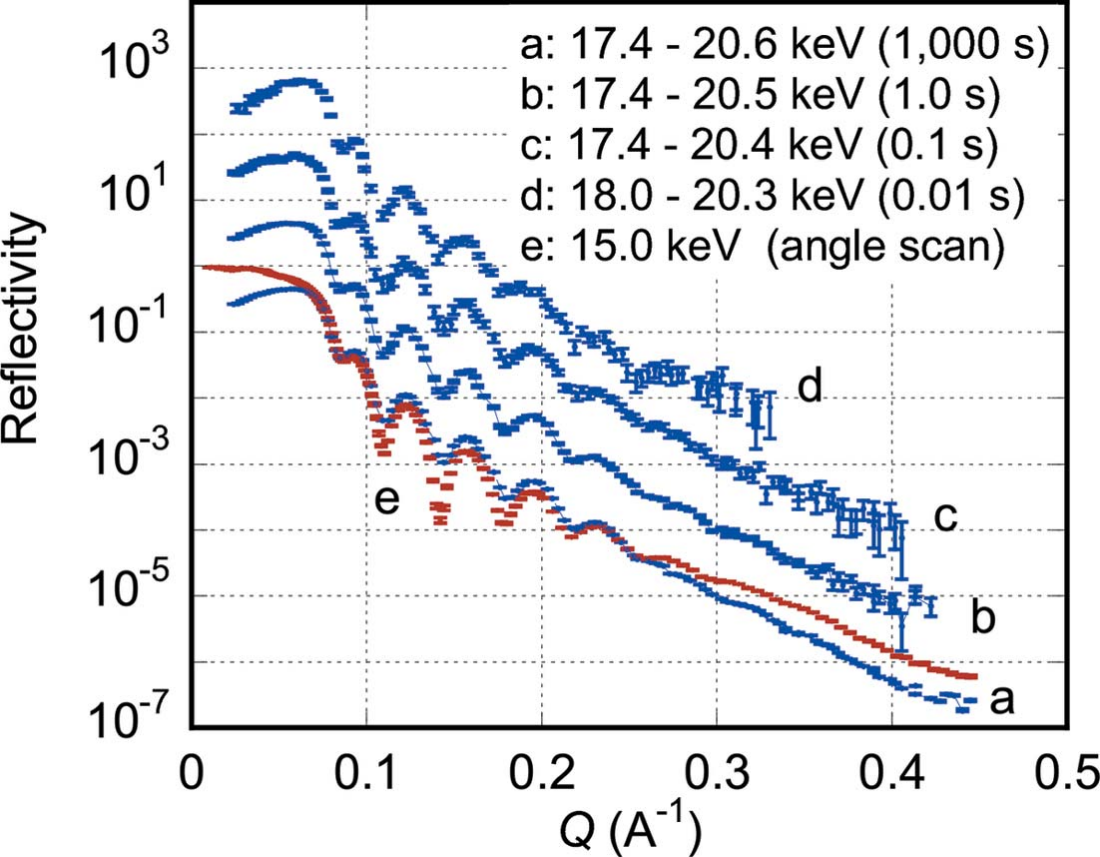
X-ray reflectivity curves from a 15.4 nm-thick gold film on a silicon single-crystal substrate. Curve *a* was obtained with a data collection time of 1000 s. Curves *b*, *c* and *d* were obtained with data collection times of 1.0, 0.1 and 0.01 s, respectively. Curves *b*, *c* and *d* are shifted vertically to avoid overlapping each other. The curve *e* was obtained with an angle-scan X-ray (*E* = 15 keV) reflectometer on an undulator beamline at SPring-8 in 644 s. The origin of its vertical axis is taken to be the same as that of the curve *a*. The used X-ray energy ranges are given in the figure.

Fig. 6 ▶ shows measured reflectivity curves from the 15.4 nm-thick gold film on a silicon substrate. Curve *a* was obtained with a data collection time of 1000 s. Curves *b*, *c* and *d* were obtained with data collection times of 1.0, 0.1 and 0.01 s, respectively. The central glancing angle of the convergent beam was made at 0.56° covering the glancing angle range 0.08–1.22° when curve *a* was measured. Curve *e* was obtained with an angle-scan X-ray (*E* = 15 keV) reflectometer (Yano *et al.*, 2009 ▶) on an undulator beamline at SPring-8 in 644 s and the origin of its vertical axis is taken to be the same as that of curve *a*.

### 4.3. Reflectivity curve from a liquid surface

**Figure 7:**
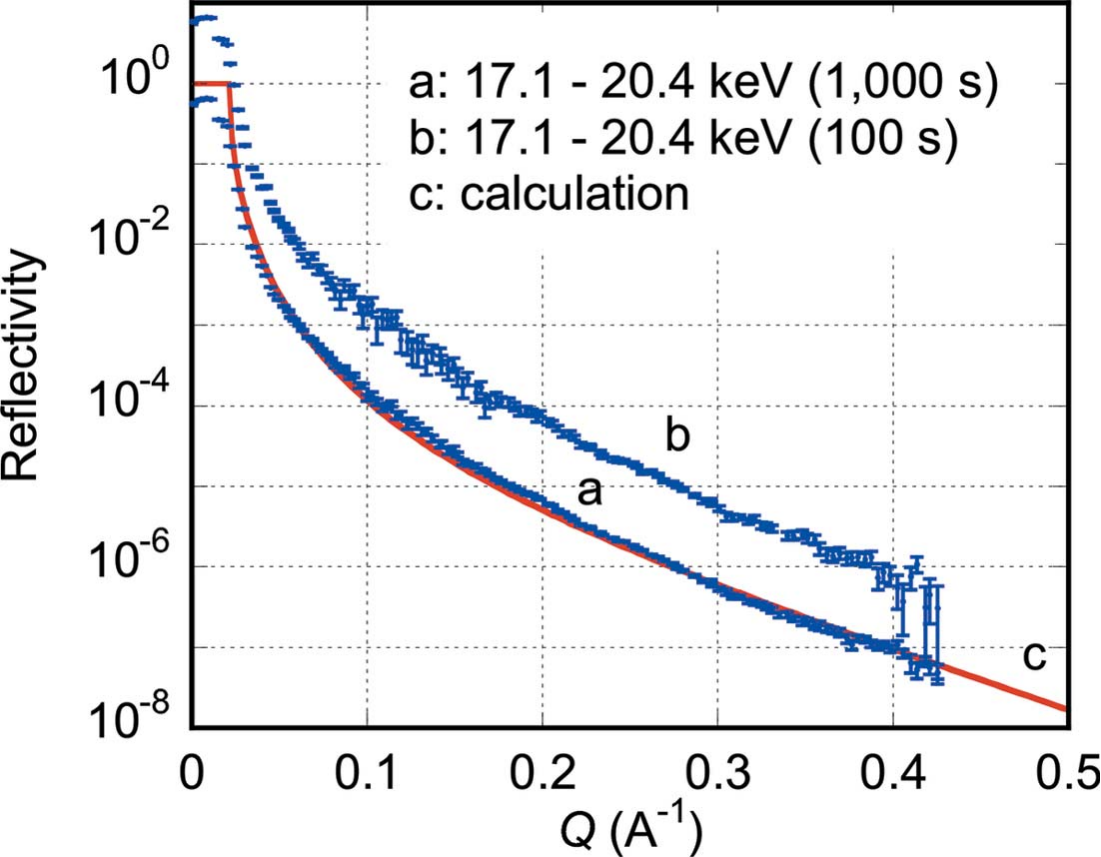
X-ray reflectivity curves from a liquid surface of ethylene glycol. Curves *a* and *b* were obtained with data collection times of 1000 and 100 s, respectively. Curve *b* is vertically shifted for clarity by one order of magnitude. The used X-ray energy ranges are given in the figure. Curve *c* is a calculated one.

Curves *a* and *b* in Fig. 7 ▶ are reflectivity curves from an ethylene glycol liquid surface obtained with data collection times of 1000 and 100 s, respectively. The simultaneously covered range of the momentum transfer was from 0.003 to 0.43 Å^−1^. The minimum reflectivity in curve *a* is 4.8 × 10^−8^ at *Q* = 0.43 Å^−1^. Curve *c* is a calculated reflectivity curve which was obtained in the same manner as in a previous paper (Yano *et al.*, 2010 ▶). Curve *b* is one order of magnitude shifted upward along the *y*-axis for clarity.

## 5. Discussion

### 5.1. Width of the simultaneously covered range and resolution of the momentum transfer

Specular X-ray reflectivity was simultaneously measured from almost zero momentum transfer to over 0.4 Å^−1^ in most curves for the silicon (100) wafer, thin gold film and liquid ethylene glycol samples. The simultaneously covered *Q*-range, Δ*Q*, is widened if we select a higher X-ray energy since Δ*Q* is proportional to the X-ray energy *E*. However, the sensitivity of the PILATUS detector falls off rapidly at high energies. As a compromise, we used X-ray energies around 20 keV in the present study. Δ*Q* would also be widened by choosing a geometry that would widen the angle range Δα although it is necessary to confirm that sufficient X-ray intensities are available in this angular range.

The largest factor contributing to the resolution of the momentum transfer is the angular width accepted by each pixel element as discussed in §2.2. This angular width was 0.0084° since Δα = 1.21° and *n* = 141 for the case of the silicon sample, which gives a resolution δ*Q*/*Q* = 0.0092 at *Q* = 0.3 Å^−1^. The resolution will be further improved by increasing the pixel number *n* in (5) using a detector of smaller pixel elements or a larger detector at a larger distance from the sample.

### 5.2. Comparison with the angle-scan method

For the case of the silicon wafer, curve *a* in Fig. 5 ▶ measured with the present dispersive method agrees very well with curve *e* measured with the angle-scan mode down to a reflectivity of ∼2 × 10^−7^. The reason for the small difference between curves *a* and *e* below a reflectivity of ∼2 × 10^−7^ is not yet clear.

For the case of a gold film on a silicon substrate, curve *a* in Fig. 6 ▶ measured with the present dispersive method agrees fairly well with curve *e* measured with the angle-scan method down to a reflectivity of ∼3 × 10^−5^. The period of the Kiessig fringes of curve *a* agrees well with that of curve *e*. The fitted thickness of the gold layer was 15.37–15.26 nm for exposure durations ranging from 1000 s to 0.1 s, while that estimated from the angle-scan curve was 15.39 nm. We assumed a single gold layer on a silicon substrate. The analysis was made after Parratt’s recursion formula (Parratt, 1954 ▶) by taking into account the energy and the glancing angle change over *Q*-value. The dips of curve *a* are shallower than those of curve *e* This is because the resolution is several times poorer in the present dispersive reflectometer than in the angle-scan reflectometer used at SPring-8. A closer comparison reveals that the reflectivity measured with the present dispersive method is slightly smaller at *Q* > 0.27 Å^−1^ than that with the angle-scan method, but the reason for this difference is not yet clear. The sample surface looked inhomogeneous and slightly different parts of the sample surface may have been irradiated by X-rays between measurements of the present dispersive and the angle-scan methods.

### 5.3. Influence of diffuse scattering to the background intensity

At a particular point (*x*_i_, *y*_i_) of the detector surface, in addition to the coplanar (within the scattering plane) diffuse scattering of X-rays incident with a glancing angle α_i_, a sum of the non-coplanar (out of the scattering plane) diffuse scattering of X-rays incident on the sample with a glancing angle α_j_ over the angle range from α_L_ to α_H_ is also recorded in the present simultaneous multiple angle-wavelength dispersive geometry. The sum of these coplanar and non-coplanar diffuse scattering overlaps with the speculary reflected X-rays and forms the background. On the other hand, in the conventional angle-scan method, only the coplanar diffuse scattering of X-rays incident with a glancing angle α_i_ overlaps with the specularly reflected beam and forms the background. For the case of the silicon (100) wafer, we compared the intensity profiles along the *y*-axis of the PILATUS detector of curve *a* (the present dispersive method) in Fig. 5 ▶ with that of curve *e* (the angle-scan method with the 0.1 mm-wide slit). In the case of the present dispersive method, the signal-to-background (S/B) ratio was relatively large (S/B > 30) at low *Q*, but medium (S/B < ∼10) or less at high *Q* (> ∼0.4 Å^−1^). On the other hand, in the angle-dispersive method using a fine slit, the S/B ratio was larger than 80–90 even at high *Q*. This means that the sum of non-coplanar diffuse scattering originating from different *Q* mainly contributes to the background intensity in the present dispersive geometry. Still, we were able to identify the specularly reflected beam surrounded by the broad and slowly varying background, as shown in Fig. 4 ▶, at levels of the specular reflectivity in the 10^−8^ range. The broad and slowly varying background intensity distribution might be partly due to the averaging effect over different glancing angles α_j_ of the X-rays and partly due to the nature of the samples used in the present study. We separated the specularly reflected beam intensity from the background intensity using the intensity profile along the *y*-axis of the image recorded by the detector. We also confirmed that the background intensity level of the intensity plot parallel to the *x*-axis is the same as that along the *y*-axis within the statistical errors and that the specular beam intensity derived by subtracting the background in the plot along the *x*-axis of the detector gives almost the same reflectivity curves within the error bars.

The present way of handling the background intensity can be compared with the case of a simultaneous multi-angle-dispersive X-ray reflectometer using a laboratory X-ray source, a knife edge and a one-dimensional detector (Naudon *et al.*, 1989 ▶). Agnihotori & Ortega (2001 ▶) reported that the reflectivity measured with the Naudon-type reflectometer is about five to ten times higher than that measured with the conventional angle-scan reflectometer even in the reflectivity range 10^−4^–10^−5^. It was difficult to separate the specular reflection intensity from the diffuse scattering intensity in their geometry. On the other hand, in the present study the specular reflection intensity is separated from the background and the agreement between reflection curves of the present dispersive method and the angle-scan method is very good down to a reflectivity of ∼2 × 10^−7^ for the case of the silicon single crystal and ∼3 × 10^−5^ for the case of the gold film.

The present procedure of subtracting the background intensity can be applied to such samples giving a relatively uniform background intensity distribution. For samples which will give a highly non-uniform background intensity distribution, a more careful background-subtraction process would be required such as two-dimensional intensity mapping around the specular reflection. This problem is a subject to be studied in the future.

The measurable minimum reflectivity is lower for less diffusely scattering material. The measured minimum reflectivity was 6.3 × 10^−9^ for the silicon wafer, while that for the ethylene glycol was 4.8 × 10^−8^.

### 5.4. Sample space

It is important that there is enough space around the sample to install chambers or equipment for controlling sample environments or applying a stimulus to the sample. In the present geometry the distance from the focus to both the upstream and downstream slits (not shown in Fig. 1 ▶) was 100 mm, giving a free space of cylindrical shape of 200 mm diameter around the sample. With a more careful alignment of these slits the diameter of this cylinder-shaped free space could be widened to 300 mm.

### 5.5. Reflectivity curves from a liquid surface

In Fig. 7 ▶ the agreement between the experimental curve *a* and the calculated curve *c* is fairly good, although there are small discrepancies in the *Q*-range of 0.08–0.22 Å^−1^. The S/B ratios in this *Q*-range were approximately 1.1–1.4, because the signal intensity *I* = *I*_0_ × *R* was very weak as a result of low values of both *I*_0_ and *R* as explained in Fig. 3 ▶. Such low values of S/B could possibly be responsible for the discrepancies. S/B ratios will be improved by increasing the X-ray intensity in this *Q*-range.

The reflectivity curves in Fig. 7 ▶ demonstrate that the present reflectometer is suitable for studying liquid surfaces owing to its characteristics that no mechanical movements of the polychromator crystal, slits, sample and detector are required during the measurement while the sample surface is kept in the horizontal plane.

### 5.6. Potential for time-resolved measurements

Since a function to successively record images is already installed in the control software of the PILATUS 100K detector with a 2.8 ms readout time for each image, time-resolved data collection will easily be conducted with a time resolution of several milliseconds or longer depending on the speed of the structural change of the sample and the X-ray intensity to be measured. Although some example curves shown in Figs. 5 ▶, 6 ▶ and 7 ▶ were obtained in seconds or sub-seconds, millisecond time resolution will be attained by using focusing mirrors and/or a more intense X-ray source such as multipole wigglers. Especially, the present method will be most suitable for samples which undergo irreversible structural changes because the time-dependent change of the whole profile of the X-ray reflectivity curves can be recorded. If the structural changes are repeatable many times upon a certain stimulus, the pump–probe method could also be applied with the present dispersive method enabling much higher time resolutions.

## 6. Summary

The performance of a simultaneous multiple angle-wavelength dispersive X-ray reflectometer was demonstrated, which can measure the whole profile of a specular X-ray reflectivity curve with no need for rotation of the sample, detector or monochromator (polychromator) crystal during the measurement. In this reflectometer a bent-twisted polychromator crystal is used to produce a convergent X-ray beam having a continuously varying energy (wavelength) and glancing angle to the sample surface as a function of direction. This X-ray beam is incident onto the sample placed horizontally at the focal point. The reflected beam intensity distribution across the beam direction was measured with a two-dimensional detector downstream of the specimen.

Examples of static measurements from a silicon single-crystal wafer and a thin gold layer on a silicon substrate suggest that reflectivity curves with the lowest reflectivity in the range of 10^−8^ can be obtained with a data collection time of 1.0–10 s. Such a time resolution will be useful in studying slow irreversible changes of surface structures. Milliseconds time resolution will be achieved if the lowest reflectivity of the reflectivity curve remains at 10^−5^ or 10^−6^ in the required range of perpendicular momentum transfer. Higher time resolutions would be attained by using more intense X-ray sources such as multi-pole wigglers at third-generation synchrotron radiation sources.

The example of ethylene glycol suggested that the present reflectometer would be suitable for studying liquid surfaces since no mechanical movement of the sample and the detector are required during measurements.

In conclusion, the present X-ray reflectometer in the simultaneous multiple angle-wavelength dispersive mode paves the way for time-resolved X-ray reflectometry of various surface structural changes, especially irreversible ones.
